# Mouse-Hamster Chimeric Prion Protein (PrP) Devoid of N-Terminal Residues 23-88 Restores Susceptibility to 22L Prions, but Not to RML Prions in PrP-Knockout Mice

**DOI:** 10.1371/journal.pone.0109737

**Published:** 2014-10-16

**Authors:** Keiji Uchiyama, Hironori Miyata, Masashi Yano, Yoshitaka Yamaguchi, Morikazu Imamura, Naomi Muramatsu, Nandita Rani Das, Junji Chida, Hideyuki Hara, Suehiro Sakaguchi

**Affiliations:** 1 Division of Molecular Neurobiology, The Institute for Enzyme Research (KOSOKEN), The University of Tokushima, Kuramato, Tokushima, Japan; 2 Animal Research Center, School of Medicine, University of Occupational and Environmental Health, Yahatanishi, Kitakyushu, Japan; 3 Influenza and Prion Disease Research Center, National Institute of Animal Health, Tsukuba, Ibaraki, Japan; Nagasaki University Graduate School of Biomedical Sciences, Japan

## Abstract

Prion infection induces conformational conversion of the normal prion protein PrP^C^, into the pathogenic isoform PrP^Sc^, in prion diseases. It has been shown that PrP-knockout (*Prnp^0/0^*) mice transgenically reconstituted with a mouse-hamster chimeric PrP lacking N-terminal residues 23-88, or Tg(MHM2Δ23-88)/*Prnp^0/0^* mice, neither developed the disease nor accumulated MHM2^Sc^Δ23-88 in their brains after inoculation with RML prions. In contrast, RML-inoculated Tg(MHM2Δ23-88)/*Prnp^0/+^* mice developed the disease with abundant accumulation of MHM2^Sc^Δ23-88 in their brains. These results indicate that MHM2Δ23-88 itself might either lose or greatly reduce the converting capacity to MHM2^Sc^Δ23-88, and that the co-expressing wild-type PrP^C^ can stimulate the conversion of MHM2Δ23-88 to MHM2^Sc^Δ23-88 *in trans*. In the present study, we confirmed that Tg(MHM2Δ23-88)/*Prnp^0/0^* mice remained resistant to RML prions for up to 730 days after inoculation. However, we found that Tg(MHM2Δ23-88)/*Prnp^0/0^* mice were susceptible to 22L prions, developing the disease with prolonged incubation times and accumulating MHM2^Sc^Δ23-88 in their brains. We also found accelerated conversion of MHM2Δ23-88 into MHM2^Sc^Δ23-88 in the brains of RML- and 22L-inoculated Tg(MHM2Δ23-88)/*Prnp^0/+^* mice. However, wild-type PrP^Sc^ accumulated less in the brains of these inoculated Tg(MHM2Δ23-88)/*Prnp^0/+^* mice, compared with RML- and 22L-inoculated *Prnp^0/+^* mice. These results show that MHM2Δ23-88 itself can convert into MHM2^Sc^Δ23-88 without the help of the *trans*-acting PrP^C^, and that, irrespective of prion strains inoculated, the co-expressing wild-type PrP^C^ stimulates the conversion of MHM2Δ23-88 into MHM2^Sc^Δ23-88, but to the contrary, the co-expressing MHM2Δ23-88 disturbs the conversion of wild-type PrP^C^ into PrP^Sc^.

## Introduction

Prions are causative agents of transmissible spongiform encephalopathies, or prion diseases, a group of fatal neurodegenerative disorders, which include Creutzfeldt-Jakob disease in humans and scrapie and bovine spongiform encephalopathy in animals [1], [2]. It is believed that prions mainly consist of the abnormally folded, relatively proteinase K (PK)-resistant pathogenic isoform of prion protein, designated PrP^Sc^ [1], [2]. PrP^Sc^ is produced by conformational conversion of the normal cellular isoform of PrP, PrP^C^, via intermolecular interaction of both molecules [1], [2]. PrP^C^ is a membrane glycoprotein tethered to the cell surface via a glycosylphosphatidylinositol moiety and expressed most abundantly in the central nervous system, particularly by neurons [3], [4]. We and others have shown that mice devoid of PrP^C^ (*Prnp^0/0^*) are resistant to prions, neither developing the disease nor producing PrP^Sc^ or propagating the prions even after inoculation with the prions [5], [6], [7], [8]. These results indicate that the conversion of PrP^C^ into PrP^Sc^ is an essential event in the pathogenesis of prion disease.

A reverse genetic approach using reconstituted *Prnp^0/0^* mice with transgenes encoding various deletion mutants of PrP^C^ is useful to investigate the structure-function relationship of PrP^C^ conversion to PrP^Sc^. *Prnp^0/0^* mice expressing mouse PrP with N-terminal residues 23-88 deleted, or Tg(PrPΔ23-88)/*Prnp^0/0^* mice, developed prion disease after inoculation with RML scrapie prions, with accumulation of PrP^Sc^Δ23-88 in their brains [9]. However, onset of the disease was delayed and the conversion of PrPΔ23-88 into PrP^Sc^Δ23-88 was inefficient [9]. These results indicate that the N-terminal residues affect the conversion of PrP^C^ into PrP^Sc^. Delayed onset of the disease was also observed in Tg(MHM2)/*Prnp^0/0^* mice inoculated with RML prions [9]. MHM2 is a mouse (M)-hamster (H) chimeric PrP, with hamster PrP-derived methionine residues at 108 and 111 instead of leucine and valine residues of mouse PrP, indicating that the chimeric region also affects the conversion. MHM2Δ23-88 is chimeric MHM2 with the deletion of N-terminal residues 23-88. Interestingly, Tg(MHM2Δ23-88)/*Prnp^0/0^* mice were reported to remain healthy for more than 500 days after inoculation with RML prions [9], [10]. No MHM2^Sc^Δ23-88 was accumulated in their brains [9], [10]. In contrast, MHM2^Sc^Δ23-88 was easily detectable in the brains of RML-inoculated Tg(MHM2Δ23-88)/*Prnp^0/+^* mice [10]. These results indicate that MHM2Δ23-88 itself might either lose or greatly reduce the converting capacity to MHM2^Sc^Δ23-88, and that the co-expressing wild-type PrP^C^ can stimulate the conversion of MHM2Δ23-88 to MHM2^Sc^Δ23-88 *in trans*.

In the present study, we confirmed that Tg(MHM2Δ23-88)/*Prnp^0/0^* mice remained resistant to RML prions for more than 730 days after inoculation. Neither MHM2^Sc^Δ23-88 nor prion infectivity was detected in their brains. However, we found that Tg(MHM2Δ23-88)/*Prnp^0/0^* mice were susceptible to 22L scrapie prions, developing prion disease around ∼530 days after inoculation. MHM2^Sc^Δ23-88 and prion infectivity were detected in the brains of terminally ill Tg(MHM2Δ23-88)/*Prnp^0/0^* mice. These results clearly demonstrate that MHM2Δ23-88 can convert to MHM2^Sc^Δ23-88 without the help of the co-expressing wild-type PrP^C^. We also found that the conversion of MHM2Δ23-88 into MHM2^Sc^Δ23-88 was accelerated and the conversion of wild-type PrP^C^ into PrP^Sc^ was contrarily decelerated in the brains of RML- and 22L-inoculated Tg(MHM2Δ23-88)/*Prnp^0/+^* mice. These results indicate that the co-expressing wild-type PrP^C^ stimulates the conversion of MHM2Δ23-88 into MHM2^Sc^Δ23-88, but to the contrary, the co-expressing MHM2Δ23-88 disturbs the conversion of wild-type PrP^C^ into PrP^Sc^, irrespective of prion strains inoculated.

## Materials and Methods

### Ethics statements

The Ethics Committee of Animal Care and Experimentation of the University of Occupational and Environmental Health, Kitakyushu, Japan, approved this study (approval number AE08-013). Animals were cared for in accordance with The Guiding Principle for Animal Care and Experimentation of the University of Occupational and Environmental Health and Japanese Law for Animal Welfare and Care.

### Animals

Tg(MHM2Δ23-88)/*Prnp^0/0^* mice with the C57BL/6×129Sv×FVB mixed background were produced elsewhere [11]. Tg(MHM2Δ23-88)/*Prnp^0/+^* and *Prnp^0/+^* mice [12] were obtained by mating Tg(MHM2Δ23-88)/*Prnp^0/0^* mice with C57BL/6 mice (CLEA Japan, Tokyo, Japan). ICR mice were purchased from Charles River Laboratories, Japan.

### Prion inoculation

Brains were removed from terminally ill C57BL/6 mice infected with RML or 22L prions. A single brain was homogenized (10%, w/v) in phosphate-buffered saline (PBS) by passing it through 18 to 26 gauge needles and then diluted to 1% with PBS. Four to five week-old mice were intracerebrally inoculated with a 20 µl-aliquot of the homogenates.

### Prion titration

10% (w/v) brain homogenates of RML- or 22L-infected, terminally ill C57BL/6 mice were serially diluted 10-fold with PBS, ranging from 10^−1^ to 10^−6^ in PBS, and a 20 µl-aliquot of 10^−1^-, 10^−4^-, 10^−5^-, and 10^−6^-diluted homogenates was intracerebrally inoculated into C57BL/6 mice aged 4–5 weeks. The mice were observed until 1 year after inoculation. The ID_50_/gram of the tissue was determined according to the method of Reed and Muench [13]. The data were statistically analyzed using logistic regression analysis.

### Western blotting

Tissue homogenates (10%, w/v) were prepared in lysis buffer containing 150 mM NaCl, 50 mM Tris-HCl (pH 7.5), 0.5% Triton X-100, 0.5% sodium deoxycholate, 1 mM EDTA, and protease inhibitor mixture (Nakalai Tesque Co., Kyoto, Japan) using a Multi-beads shocker (Yasui Kikai Co., Osaka, Japan). Protein concentrations were determined using the BCA protein assay kit (Pierce, Rockford, USA.). Total proteins were treated with or without PK (Wako Pure Chemical Industries, Ltd., Osaka, Japan) at 20 µg/ml for 30 min at 37°C, electrophoresed through an SDS-polyacrylamide gel, and electrically transferred to an Immobilon-P PVDF membrane (Millipore Corp., MA, USA). The membrane was immersed in 5% non-fat dry milk-containing TBST (0.1% Tween-20, 100 mM NaCl, 10 mM Tris-HCl, pH 7.6) for 1 h at room temperature (RT), and incubated with M20 goat polyclonal antibodies (Santa Cruz Biotechnology, Inc., Santa Cruz, CA), 3F4 monoclonal antibody (Signet Laboratories Inc., Dedham, MA), or anti-β-actin monoclonal antibody (Sigma-Aldrich, Inc., St. Louis, MO) for 2 h at RT or overnight at 4°C in 1% non-fat dry milk-containing TBST. The membrane was washed in TBST several times. Signals were visualized using horseradish peroxidase-conjugated anti-mouse IgG antibodies (Amersham Biosciences Inc., Piscataway, NJ) and anti-goat IgG antibodies (CHEMICON International, Inc., Temecula, CA) and Immobilon Western Chemiluminescent HRP substrate (Millipore), and then detected using a chemiluminescence image analyzer, LAS-4000 mini (Fujifilm Co., Tokyo, Japan).

### Hematoxylin-eosin staining

Paraffin-embedded samples were sectioned at 5 µm. The sectioned samples were deparaffinized, rehydrated, and stained with Mayer's hematoxylin solution (Wako Pure Chemical Industries, Ltd., Osaka, Japan) and 1% Eosin Y solution (Wako Pure Chemical Industries, Ltd.). After washing, the samples were mounted with Softmount (Wako Pure Chemical Industries, Ltd.).

### Vector constructions

The DNA fragment encoding mouse PrP residues 104–254 with methionine substitutions at codons 108 and 111 was first amplified by polymerase chain reaction (PCR) with a sense primer (5′-ccaaaaaccaacatgaagcaca-3′) and an antisense primer (5′-cctctagacc**tca**tcccacgatcaggaaga-3′; the underlined sequence is an *Xba* I site; the bold sequence is a stop codon) using pcDNA3.1-moPrP plasmid [14] as a template. The resulting DNA fragment was then utilized as a 3′ primer together with a sense primer (5′-tcggatccagtcatc**atg**gcgaaccttggc-3′; the underlined sequence is a *Bam*H I site; the bold sequence is a start codon) to amplify a DNA fragment encoding full-length mouse PrP with the methionine substitutions using the pcDNA3.1-moPrP template plasmid. After confirmation of the DNA sequences, the DNA fragment was digested by *BamH* I and *Xba* I and introduced into a pcDNA3.1 vector (Invitrogen, Carlsbad, CA) to generate pcDNA3.1-MHM2. Finally, to construct *E. coli* expression vectors for recombinant full-length MHM2 and MHM2Δ23-88, the corresponding DNA fragments were amplified by Phusion High-Fidelity DNA Polymerase (Thermo Scientific, Waltham, MA) using pcDNA3.1-MHM2 plasmid template with sense primers (5′-ctcggatcc *aaaaagcggccaaagcctgga*-3′ for MHM2, the underlined sequence is a *Bam*H I site; the italic sequence, residues 23–29 of mouse PrP; 5′-ctcggatcc *ggccaaggagggggtacccat*-3′ for MHM2Δ23-88, the underlined sequence is a *Bam*H I site; the italic sequence, residues 89–95 of mouse PrP) and an antisense primer (5′-ctcaagctt **tca** *tcccacgatcaggaagat*-3′, the underlined sequence is a *Hind* III site; the bold sequence is a stop codon; the italic sequence, residues 249–254 of mouse PrP). After confirmation of the DNA sequences, the DNA fragments were digested by *BamH* I and *Hind* III and introduced into a pQE30 vector (Qiagen, Hilden, Germany) to generate pQE30-MHM2 and pQE30-MHM2Δ23-88.

### Purification of recombinant proteins

*E. coli* was transformed by pQE30-MHM2 and pQE30-MHM2Δ23-88 and cultured at 37°C overnight in the presence of 1 mM isopropyl β-D-thiogalactoside to induce expression of recombinant full-length MHM2 and MHM2Δ23-88, both of which are His-tagged. The *E. coli* cells cultured in 1 L medium were harvested by centrifugation (3,000×g, 20 min, 4°C) and the resulting pellet was suspended in 30 mL of 6 M GdnHCl solution (0.1 M NaH_2_PO_4_, 0.01 M Tris, 6 M guanidine hydrochloride, pH 8.0). The cells were lysed by rotating the suspension for 30 min at RT followed by sonication for 1 min at intervals of 1 min 4 times. The suspension was again subjected to rotation for 30 min at RT and then centrifuged (12,000×g, 20 min, 25°C). The resulting supernatant was mixed with 0.5 mL (bed volume) of Ni-NAT agarose (Qiagen) and rotated for 2 h at RT. The Ni-NAT agarose was collected by centrifugation (500×g, 3 min, 25°C) and washed 3 times with 10 mL of 8 M urea solution (0.1 M NaH_2_PO_4_, 0.01 M Tris, 8 M urea, pH 6.3). To decrease the urea concentration in the suspension in a stepwise fashion, the Ni-NAT agarose pellet was suspended in 9 mL of the 8 M urea solution and 1 mL of dilution solution (0.1 M NaH_2_PO_4_, 0.01 M Tris, pH 6.3). The suspension was rotated at 4°C for 30 min and centrifuged (500×g, 3 min, 4°C). 1 mL of the resulting supernatant was removed, 1 mL of the dilution solution was instead added to the supernatant, and the diluted supernatant was rotated at 4°C for 30 min and centrifuged (500×g, 3 min, 4°C). This dilution procedure was repeated 13 times in total. After the last dilution step, the Ni-NAT agarose was washed 3 times with 10 mL of 2 M Urea solution (0.1 M NaH_2_PO_4_, 0.01 M Tris, 2 M urea, pH 6.3) containing 0.2% Triton X-100 and 3 times with 10 mL of 10 mM imidazole solution (50 mM NaH_2_PO_4_, 300 mM NaCl, 10 mM imidazole, pH 8.0) containing 0.2% Triton X-100. Finally, recombinant proteins were eluted from the Ni-NAT agarose in 5×0.4 mL of 0.5 M imidazole solution (50 mM NaH_2_PO_4_, 300 mM NaCl, 0.5 M imidazole, pH 8.0) containing 0.2% Triton X-100. The eluted proteins were further purified with a Superdex 200 gel filtration column (Life Technologies, Carlsbad, CA) with GF buffer [20 mM 4-(2-hydroxyethyl)-1-piperazineethanesulfonic acid, 0.15 M KCl, 1 mM dithiothreitol, 5% glycerol, 0.2% Triton X-100].

### Pull-down assay

Clarified brain homogenate was prepared as follows. 5% (w/v) brain homogenate in PBS was sonicated for 1 min and centrifuged at 500×g for 15 min at 4°C. The supernatant was collected and diluted with PBS to 3 µg-protein/ml. Anti-His4 ab-Protein G beads were prepared as follows. Anti-His4 antibody (Qiagen) (0.3 mg) was incubated with 10 mL of Dynabeads Protein G (Life technologies) in 200 mL of binding buffer (PBS containing 1% Triton X-100 and 1% Tween 20) at RT. After 1 h-incubation, the beads were washed with the binding buffer to remove unbound antibody. The purified His-tagged recombinant PrP was incubated with 75 µg of the clarified brain homogenate in the binding buffer containing 2 M urea at 4°C for 1 h. The anti-His4 ab-Protein G beads were added and the mixture was rotated at 4°C for 4 h. The immune complexes were collected using a magnetic separation rack and washed with wash buffer (PBS containing 0.05% Triton X-100 and 0.05% Tween 20). After washing, the complexes were incubated in 30 mL of lysis buffer (150 mM NaCl, 50 mM Tris-HCl, pH 7.5, 0.5% Triton X-100, 0.5% sodium deoxycholate, 1 mM EDTA) containing 50 µg/ml of PK at 37°C for 30 min. The PK-treated samples were subjected to Western blotting.

## Results

### Tg(MHM2Δ23-88)/*Prnp^0/0^* mice develop prion disease after inoculation with 22L prions, but not with RML prions

We intracerebrally inoculated RML and 22L prions into Tg(MHM2Δ23-88)/*Prnp^0/0^*, Tg(MHM2Δ23-88)/*Prnp^0/+^*, and *Prnp^0/+^* mice. We previously reported that *Prnp^0/0^* mice used in this study developed cerebellar ataxia at 469±46 days after birth due to ectopic expression of a PrP-like molecule Dpl in their brains [12], [14], and that the ataxia was not rescued in Tg(MHM2Δ23-88)/*Prnp^0/0^* mice [11]. Therefore, to avoid false diagnosis of symptoms specifically developed in inoculated Tg(MHM2Δ23-88)/*Prnp^0/0^* mice, we reared the inoculated mice together with un-inoculated age-matched *Prnp^0/0^* mice at the same time and carefully compared the symptoms appearing in inoculated Tg(MHM2Δ23-88)/*Prnp^0/0^* mice to those in un-inoculated *Prnp^0/0^* mice. No specific symptoms other than the ataxia were observed in Tg(MHM2Δ23-88)/*Prnp^0/0^* mice more than 730 days post-inoculation (dpi) with RML prions (Table 1). In contrast, Tg(MHM2Δ23-88)/*Prnp^0/0^* mice inoculated with 22L prions developed specific symptoms, such as decreased locomotion, urinary incontinence, ruffled body hair and, in some male cases, priapism with incubation times at 538±29 dpi (Table 1). Tg(MHM2Δ23-88)/*Prnp^0/+^* and *Prnp^0/+^* mice also developed the disease at 347±16 and 393±13 dpi with RML prions and at 277±2 and 272±1 dpi with 22L prions, respectively (Table 1). We repeated the same experiments with increased numbers of mice and obtained consistent results (Table 1).

**Table 1:** Incubation times of different mouse lines intracerebrally inoculated with RML and 22L prions.

	Mouse lines	Protein expression levels^a^		Incubation times (average ± standard deviation in days)	
		Full-length PrP^C^	Mutant PrP	RML	22L
First experiment	*Prnp^0/+^*	0.5×	-	393 ± 13 (7/7)^b^	272±1 (5/5)
	MHM2Δ23-88/*Prnp^0/+^*	0.5×	4×	347±16 (6/6)	277±2 (7/7)
	MHM2Δ23-88/*Prnp^0/0^*	-	4×	>730 (5/5)	538±29 (3/3)
Second experiment	*Prnp^0/+^*	0.5×	-	387±14 (13/13)	261±1 (9/9)
	MHM2Δ23-88/*Prnp^0/+^*	0.5×	4×	365±12 (14/14)	265±8 (10/10)
	MHM2Δ23-88/*Prnp^0/0^*	-	4×	>672 (6/6)	527±59 (7/7)

a Expression levels of full-length PrP^C^ or mutant PrP are compared with those of PrP^C^ in wild-type mice (ref 10).

b The numbers in parentheses indicate the number of diseased mice/the number of inoculated mice.

There is a possibility that the different susceptibility of Tg(MHM2Δ23-88)/*Prnp^0/0^* mice to RML and 22L prions might not be due to different responsiveness of MHM2Δ23-88 to RML and 22L prions, but rather due to different infectious doses of RML and 22L prions inoculated. To rule out this possibility, we determined infectious titers in the brain homogenates from RML- or 22L-infected, terminally ill C57BL/6 mice that were similarly prepared as the brain homogenates used as inocula. The serially diluted brain homogenates were intracerebrally inoculated into C57BL/6 mice. No significant difference was detected in the number of diseased mice between the groups inoculated with each dilution of RML- and 22L-infected brain homogenates (Logistic regression analysis, p = 0.083, Table 2), indicating that infectious titers are not different in the RML- and 22L-infected brain homogenates used (Table 2). According to the method of Reed and Muench [13], ID_50_/gram of the brain tissue was calculated as 10^7.7^ and 10^8.2^ in the RML- and 22L-infected brain homogenates, respectively (Table 2). These results indicate that the different susceptibility of Tg(MHM2Δ23-88)/*Prnp^0/0^* mice to RML and 22L prions is due to different responsiveness of MHM2Δ23-88 to RML and 22L prions. C57BL/6 mice inoculated with RML and 22L prions developed the disease with similar incubation times (Table 2). However, RML-inoculated *Prnp^0/+^* mice showed longer incubation times than 22L-inoculated *Prnp^0/+^* mice (Table 1). These results suggest that RML and 22L prions are affected in their propagation by the expression levels of PrP^C^, with RML prions being affected more strongly than 22L prions.

**Table 2:** Titration of the brain homogenates of RML- or 22L-infected terminally ill C57BL/6 wild-type mice.

Dilution of brain homogenates	Incubation times^b^ (No. of diseased mice/No. of inoculated mice)	
	RML	22L
10^−1^	135±1 (6/6)	133±2 (6/6)
10^−4^	220±14 (4/5)	228±15 (5/5)
10^−5^	251±49 (3/6)	262±31 (5/6)
10^−6^	>365 (0/6)	308 (1/6)
Infectious titers (ID_50_/gram of tissue)^a^	10^7.7^	10^8.2^

a ID_50_/gram of the tissue was calculated by the method of Reed and Muench [13].

b Incubation times are indicated as average ± standard deviation (days).

**Figure 1:**
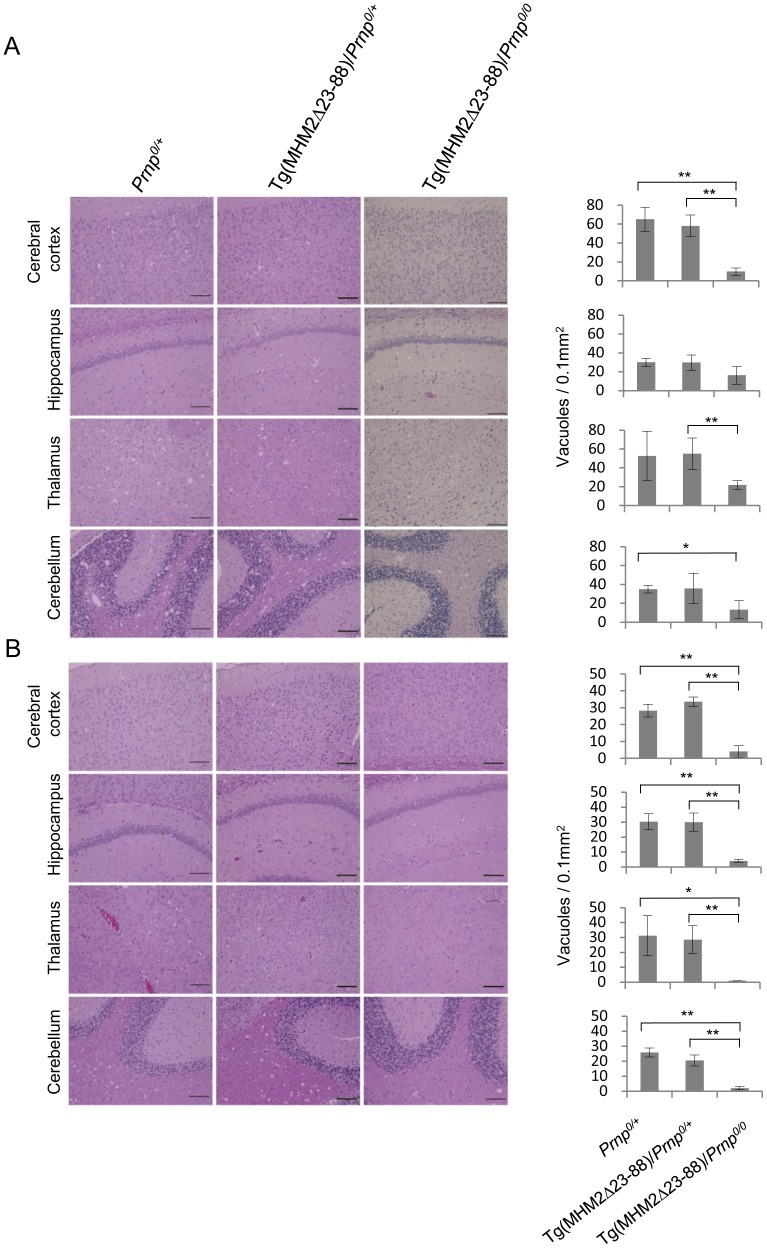
HE-stained brain sections from different genotypic mice inoculated with prions. (A) Spongiosis is milder in the cerebral cortex, hippocampus, thalamus and cerebellum from 22L-inoculated, terminally ill Tg(MHM2Δ23-88)/*Prnp^0/0^* mice than in 22L-inoculated, terminally ill *Prnp^0/+^* and Tg(MHM2Δ23-88)/*Prnp^0/+^* mice. (B) Spongiosis is observed in the cerebral cortex, hippocampus, thalamus and cerebellum from RML-inoculated, terminally ill *Prnp^0/+^* and Tg(MHM2Δ23-88)/*Prnp^0/+^* mice, but not from RML-inoculated, symptom-free Tg(MHM2Δ23-88)/*Prnp^0/0^* mice. Vacuoles were counted in 0.1 mm^2^ in each brain area of different genotypic mice (n = 3–4/each genotype) and evaluated by Student's t-test. Scale bar, 100 µm. *, p<0.05; **, p<0.01.

We then investigated the brain sections from these inoculated mice for spongiosis, a pathological hallmark of prion disease. The sections were subjected to hematoxylin-eosin staining. Spongiosis was easily detectable in the brains of 22L-inoculated, terminally ill Tg(MHM2Δ23-88)/*Prnp^0/0^* (n = 3), Tg(MHM2Δ23-88)/*Prnp^0/+^* (n = 4), and *Prnp^0/+^* mice (n = 3), particularly in the cerebral cortex, hippocampus, thalamus, and cerebellum (Fig. 1A and S1A). It was mild in Tg(MHM2Δ23-88)/*Prnp^0/0^* mice compared to that in Tg(MHM2Δ23-88)/*Prnp^0/+^* and *Prnp^0/+^* mice (Fig. 1A and S1A). Spongiosis was also observed in the sections of RML-inoculated, terminally ill Tg(MHM2Δ23-88)/*Prnp^0/+^* (n = 4) and *Prnp^0/+^* mice (n = 4) (Fig. 1B and S1B). However, spongiosis was hardly detectable in Tg(MHM2Δ23-88)/*Prnp^0/0^* mice, which were inoculated with RML prions and sacrificed at 419 (n = 2) and 595 dpi (n = 1) (Fig. 1B and S1B). These results clearly demonstrate that Tg(MHM2Δ23-88)/*Prnp^0/0^* mice inoculated with 22L prions developed prion disease while those inoculated with RML prions were disease-free.

### MHM2^Sc^Δ23-88 is detectable in the brains of terminally ill, 22L-inoculated Tg(MHM2Δ23-88)/*Prnp^0/0^* mice

**Figure 2:**
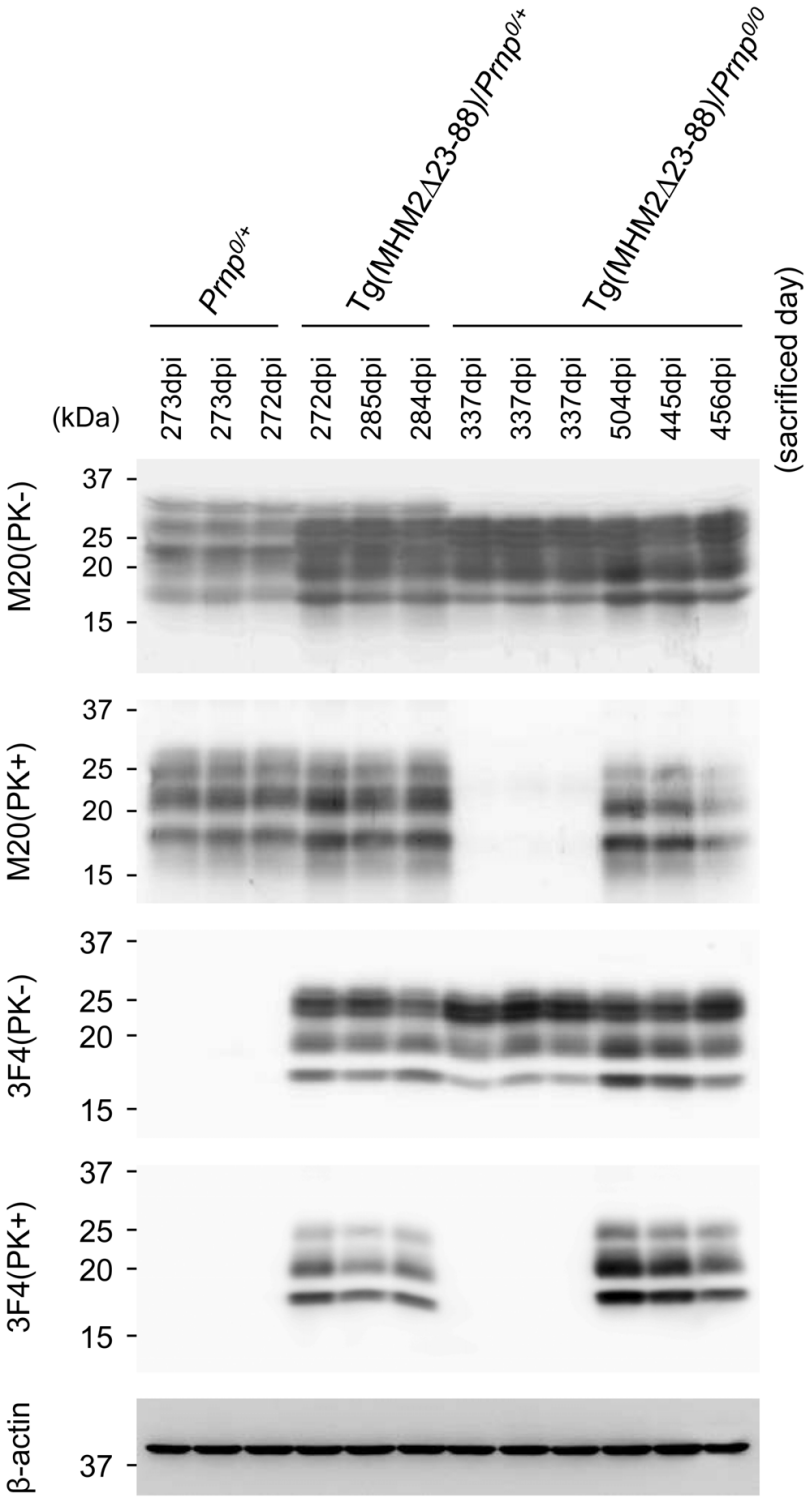
Western blotting of the brains of different genotypic mice inoculated with 22L prions. Brains from terminally ill, 22L-inoculated *Prnp^0/+^*, Tg(MHM2Δ23-88)/*Prnp^0/+^*, and Tg(MHM2Δ23-88)/*Prnp^0/0^* mice, and from asymptomatic, 22L-inoculated Tg(MHM2Δ23-88)/*Prnp^0/0^* mice (sacrificed at 337 dpi) were subjected to Western blotting using M20 and 3F4 anti-PrP antibodies after treatment with or without PK. M20 antibodies detect wild-type and mutant PrPs. 3F4 antibody recognizes only the mutant PrP. The PK-resistant MHM2Δ23-88, or MHM2^Sc^Δ23-88, is detectable in terminally ill, 22L-inoculated Tg(MHM2Δ23-88)/*Prnp^0/+^* and Tg(MHM2Δ23-88)/*Prnp^0/0^* mice, but not in asymptomatic, 22L-inoculated Tg(MHM2Δ23-88)/*Prnp^0/0^* mice. β-actin is an internal control.

**Figure 3:**
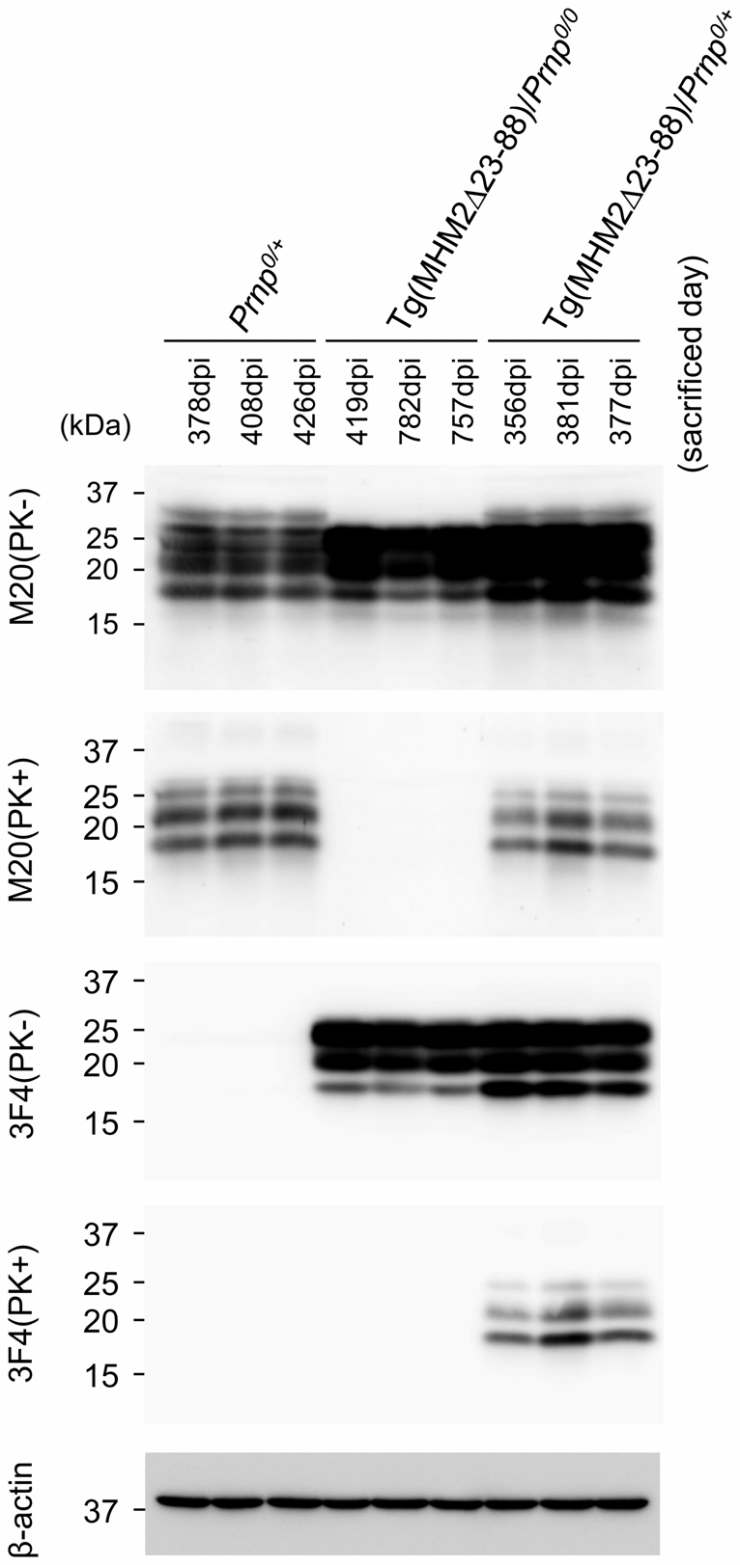
Western blotting of the brains of different genotypic mice inoculated with RML prions. Brains from terminally ill, RML-inoculated *Prnp^0/+^* and Tg(MHM2Δ23-88)/*Prnp^0/+^* mice, and from asymptomatic, RML-inoculated Tg(MHM2Δ23-88)/*Prnp^0/0^* mice (sacrificed at 419, 757, and 782 dpi) were subjected to Western blotting with M20 and 3F4 antibodies after treatment with or without PK. MHM2^Sc^Δ23-88 is detectable in Tg(MHM2Δ23-88)/*Prnp^0/+^* mice, but not in Tg(MHM2Δ23-88)/*Prnp^0/0^* mice. β-actin is an internal control.

We investigated PK-resistant PrP in the brains of 22L-inoculated Tg(MHM2Δ23-88)/*Prnp^0/0^*, Tg(MHM2Δ23-88)/*Prnp^0/+^* and *Prnp^0/+^* mice using Western blotting. MHM2Δ23-88 was reported to be overexpressed in brains about 4-times more than wild-type PrP^C^ in the hamster brain [10], thus giving rise to stronger signals for total PrP in Tg mice than in non-Tg *Prnp^0/+^* mice on Western blotting with M20 anti-PrP antibodies (Fig. 2 and 3, 1^st^ panel). M20 antibodies were raised against a C-terminal peptide of PrP, and are therefore able to detect both wild-type PrP and MHM2Δ23-88. M20 antibodies also detected PK-resistant PrP in the brains of 22L-inoculated, terminally ill Tg(MHM2Δ23-88)/*Prnp^0/0^*, Tg(MHM2Δ23-88)/*Prnp^0/+^* and *Prnp^0/+^* mice (Fig. 2, 2^nd^ panel). The PK-resistant PrP in Tg(MHM2Δ23-88)/*Prnp^0/0^* and Tg(MHM2Δ23-88)/*Prnp^0/+^* mice was recognized by 3F4 anti-PrP antibody (Fig. 2, 4^th^ panel), which is able to detect only the mutant protein (Fig. 2, 3^rd^ panel), indicating that MHM2Δ23-88 was converted into MHM2^Sc^Δ23-88 in 22L-inoculated Tg(MHM2Δ23-88)/*Prnp^0/0^* and Tg(MHM2Δ23-88)/*Prnp^0/+^* mice.

MHM2^Sc^Δ23-88 was undetectable in the brains of asymptomatic Tg(MHM2Δ23-88)/*Prnp^0/0^* mice, which were sacrificed at 337 dpi with 22L prions (Fig. 2, 4^th^ panel). In contrast, terminally ill Tg(MHM2Δ23-88)/*Prnp^0/+^* mice, which were sacrificed at 272–285 dpi with 22L prions, already accumulated MHM2^Sc^Δ23-88 in their brains (Fig. 2, 4^th^ panel). These results indicate that the conversion of MHM2Δ23-88 into MHM2^Sc^Δ23-88 was enhanced in Tg(MHM2Δ23-88)/*Prnp^0/+^* mice after inoculation with 22L prions.

### MHM2^Sc^Δ23-88 is undetectable in the brains of RML-inoculated Tg(MHM2Δ23-88)/*Prnp^0/0^* mice

We also investigated PK-resistant PrP in the brains of RML-inoculated Tg(MHM2Δ23-88)/*Prnp^0/0^*, Tg(MHM2Δ23-88)/*Prnp^0/+^* and *Prnp^0/+^* mice using Western blotting with M20 and 3F4 antibodies. Western blotting with M20 antibodies revealed PK-resistant PrP accumulated in the brains of RML-inoculated, terminally ill Tg(MHM2Δ23-88)/*Prnp^0/+^* and *Prnp^0/+^* mice (Fig. 3, 2^nd^ panel). The PK-resistant PrP in Tg(MHM2Δ23-88)/*Prnp^0/+^* mice was also detected with 3F4 antibody (Fig. 3, 4^th^ panel), indicating that MHM2Δ23-88 was converted into MHM2^Sc^Δ23-88 in Tg(MHM2Δ23-88)/*Prnp^0/+^* mice inoculated with RML prions. No PK-resistant PrP was observed in the brains of Tg(MHM2Δ23-88)/*Prnp^0/0^* mice, which were inoculated with RML prions and sacrificed 419, 755, and 782 dpi, by Western blotting with M20 and 3F4 antibodies (Fig. 3, 2^nd^ and 4^th^ panels).

### Conversion of wild-type PrP^C^ into PrP^Sc^ is reduced in the brains of Tg(MHM2Δ23-88)/*Prnp^0/+^* mice inoculated with 22L and RML prions

**Figure 4:**
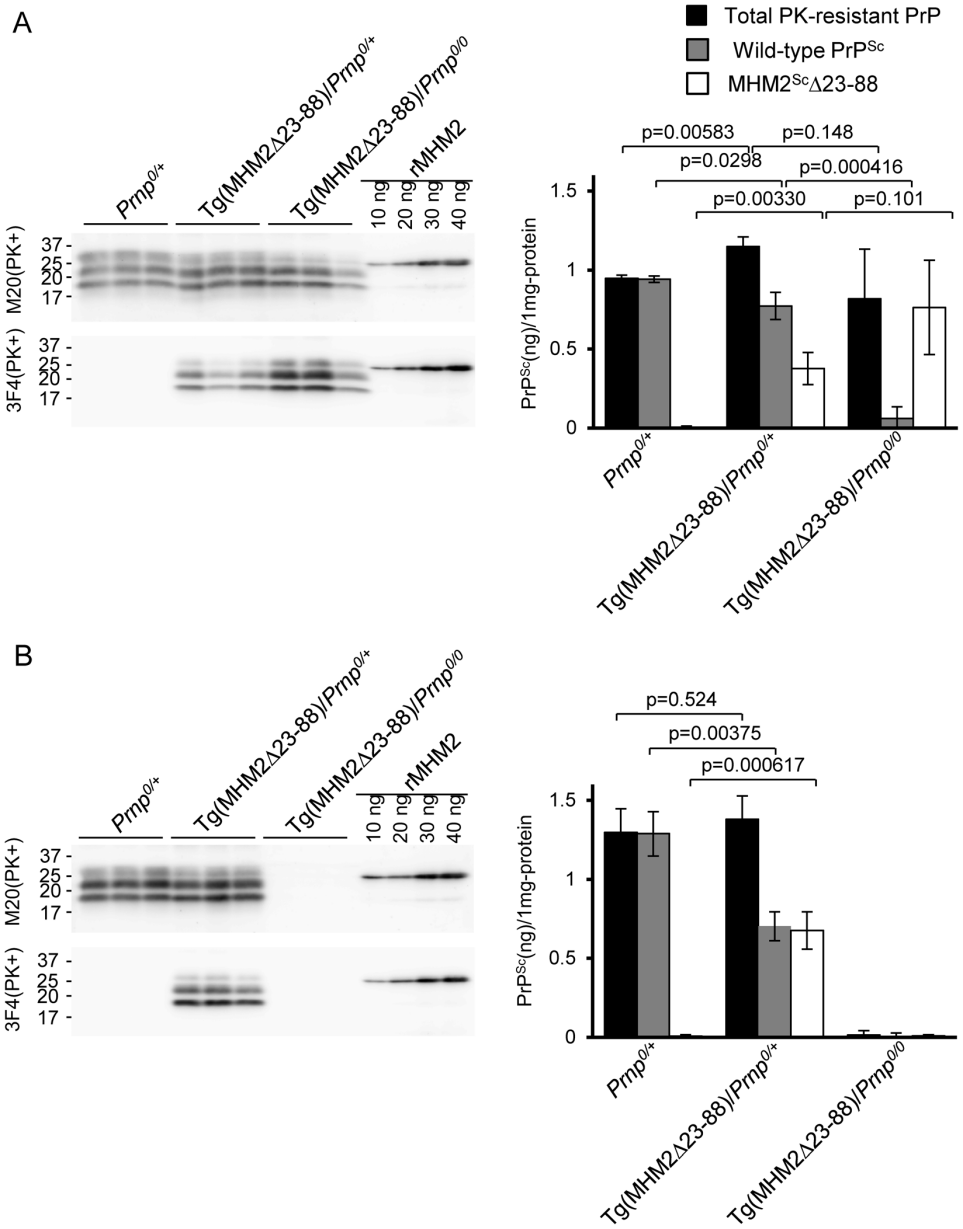
Semi-quantification of wild-type PrP^Sc^ and MHM2^Sc^Δ23-88 in brains of different genotypic mice inoculated with prions. PK-treated brain homogenates from different genotypic mice inoculated with 22L (A) and RML prions (B) were analyzed for signal densities of total PK-resistant PrP and MHM2^Sc^Δ23-88 on Western blotting with M20 and 3F4 anti-PrP antibodies, respectively. Purified recombinant MHM2 protein was used as a reference to estimate the amounts of total PK-resistant PrP and MHM2^Sc^Δ23-88. Amounts of wild-type PrP^Sc^ were calculated by subtraction of the amounts of MHM2^Sc^Δ23-88 from those of total PK-resistant PrP. Data were analyzed by Student's t-test.

We investigated whether MHM2Δ23-88 could affect the conversion of wild-type PrP^C^ into PrP^Sc^ by semi-quantifying wild-type PrP^Sc^ and MHM2^Sc^Δ23-88 accumulated in the brains of RML- and 22L-inoculated, terminally ill Tg(MHM2Δ23-88)/*Prnp^0/+^* mice. Purified recombinant MHM2 protein with a 6×His tag was used as a reference to estimate the amounts of M20-positive total PK-resistant PrP and 3F4-positive MHM2^Sc^Δ23-88. Amounts of wild-type PrP^Sc^ were calculated by subtraction of the amounts of MHM2^Sc^Δ23-88 from those of total PK-resistant PrP. As a result, the amount of wild-type PrP^Sc^ was reduced in the brains of 22L-inoculated, terminally ill Tg(MHM2Δ23-88)/*Prnp^0/+^* mice, compared to those in 22L-inoculated, terminally ill *Prnp^0/+^* mice (p = 0.0298, Fig. 4A). Wild-type PrP^Sc^ was also reduced in the brains of RML-inoculated, terminally ill Tg(MHM2Δ23-88)/*Prnp^0/+^* mice, compared to those in RML-inoculated, terminally ill *Prnp^0/+^* mice (p = 0.00375, Fig. 4B). These results indicate that, in contrast to the conversion of MHM2Δ23-88 into MHM2^Sc^Δ23-88 being enhanced, the conversion of wild-type PrP^C^ into PrP^Sc^ was decelerated in the brains of Tg(MHM2Δ23-88)/*Prnp^0/+^* mice inoculated with RML and 22L prions.

### Prion infectivity in the brains of Tg(MHM2Δ23-88)/*Prnp^0/0^* mice inoculated with 22L and RML prions

**Figure 5:**
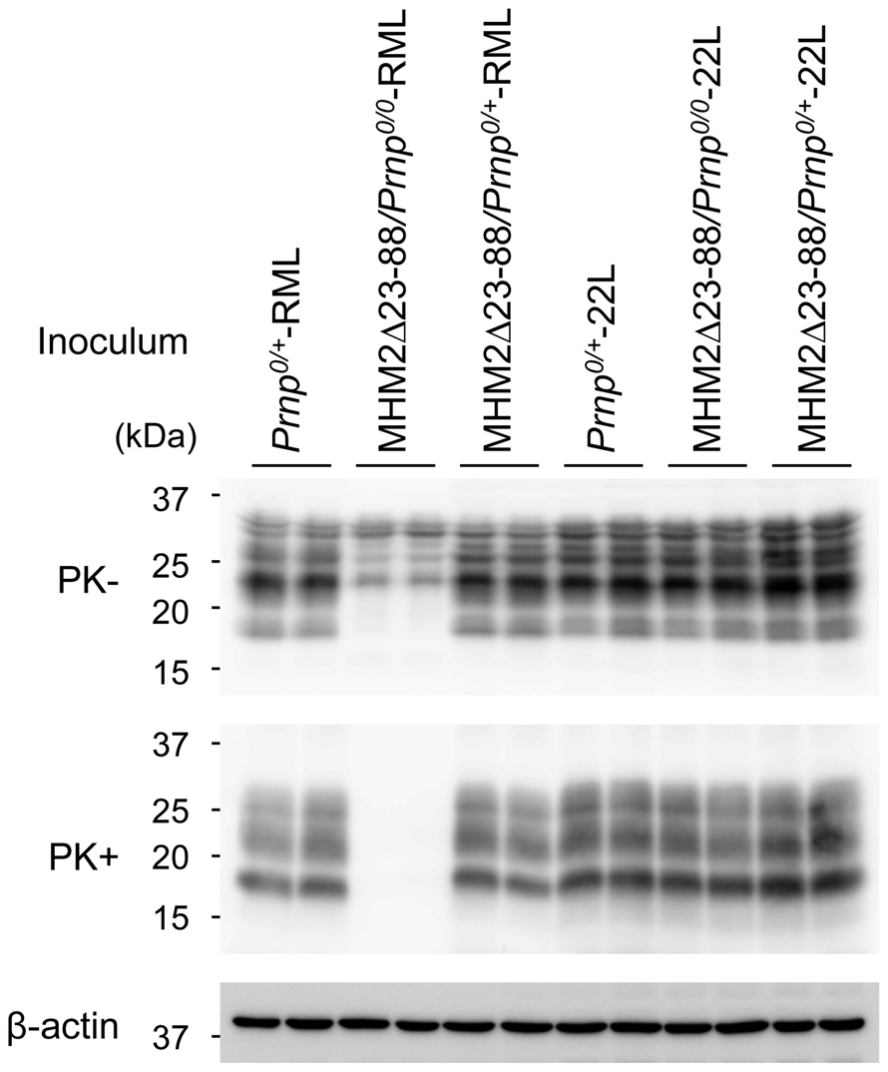
Western blotting of the brains from indicator mice inoculated with the brain homogenates from different genotypic mice inoculated with RML and 22L prions. PK-resistant PrP is detectable with M20 anti-PrP antibodies in all inoculated indicator mice, except for the mice sacrificed at 365 days after inoculation with the brain homogenate from the Tg(MHM2Δ23-88)/*Prnp^0/0^* mouse, which was sacrificed at 591 days after inoculation with RML prions.

To investigate whether the MHM2^Sc^Δ23-88-associated prions could be transmissible to wild-type mice, we intracerebrally inoculated single brain homogenate from 22L-inoculated, terminally ill Tg(MHM2Δ23-88)/*Prnp^0/0^* mouse into wild-type indicator ICR mice. Single brain homogenates from 22L-inoculated, terminally ill Tg(MHM2Δ23-88)/*Prnp^0/+^* and *Prnp^0/+^* mice were also inoculated into indicator mice. Indicator mice developed the disease at 175±10, 153±5, and 159±7 days after inoculation with the homogenates from Tg(MHM2Δ23-88)/*Prnp^0/0^*, Tg(MHM2Δ23-88)/*Prnp^0/+^*, and *Prnp^0/+^* mice, respectively (Table 3). All diseased indicator mice exhibited indistinguishable symptoms including ataxia, and accumulated PrP^Sc^, with the same PK-resistant fragments and glycosylation patterns, in their brains (Fig. 5). These results indicate that the MHM2^Sc^Δ23-88-associated prions were transmissible to wild-type mice probably with the same pathogenic properties as the original 22L prions. However, the incubation times of the indicator mice inoculated with the Tg(MHM2Δ23-88)/*Prnp^0/0^* brain homogenate were significantly longer than those inoculated with the *Prnp^0/+^* or Tg(MHM2Δ23-88)/*Prnp^0/+^* brain homogenate (Table 3), suggesting that MHM2^Sc^Δ23-88-associated prions are slightly less transmissible to wild-type mice than wild-type PrP^Sc^-associated prions.

**Table 3:** A bioassay for elucidation of prion infectivity in the brains of different mouse lines intracerebrally inoculated with RML and 22L prions.

Donor mouse lines of brain homogenate inoculum	Recipient indicator mice		
	Strain	Protein expression levels^a^	Incubation times^b^ (No. of diseased mice/No. of inoculated mice)
22L-inoculated *Prnp^0/+^*	ICR wild-type	1×	159±7 (6/6)
22L-inoculated MHM2Δ23-88/*Prnp^0/+^*	ICR wild-type	1×	153±5 (5/5)
22L-inoculated MHM2Δ23-88/*Prnp^0/0^*	ICR wild-type	1×	175±10 (5/5)*
RML-inoculated *Prnp^0/+^*	ICR wild-type	1×	147±5 (5/5)
RML-inoculated MHM2Δ23-88/*Prnp^0/+^*	ICR wild-type	1×	144±5 (5/5)
RML-inoculated MHM2Δ23-88/*Prnp^0/0^*	ICR wild-type	1×	>365 (4/4)

a Expression levels of full-length PrP^C^ in wild-type mice are estimated as 1.

b Incubation times are indicated as average ± standard deviation (days).

*Indicates a significant difference (*p* = 0.0206, Log-rank test) compared to the incubation times of indicator mice inoculated with the *Prnp^0/+^* and MHM2Δ23-88/*Prnp^0/+^* brain homogenates.

We also investigated prion infectivity in the brain of a Tg(MHM2Δ23-88)/*Prnp^0/0^* mouse, which was inoculated with RML prions and sacrificed at 591 dpi. None of the indicator mice inoculated with the single brain homogenate from the Tg(MHM2Δ23-88)/*Prnp^0/0^* mouse became sick by 365 dpi (Table 3). PrP^Sc^ was also undetectable in the brains of indicator mice sacrificed at 365 dpi (Fig. 5). In contrast, single brain homogenates from RML-inoculated, terminally ill *Prnp^0/+^* and Tg(MHM2Δ23-88)/*Prnp^0/+^* mice caused the disease in indicator mice at 147±5 and 144±5 dpi, respectively (Table 3), with abundant accumulation of PrP^Sc^ in their brains (Fig. 5). These results are consistent with MHM2^Sc^Δ23-88 being undetectable in the brains of RML-inoculated Tg(MHM2Δ23-88)/*Prnp^0/0^* mice.

### Binding of recombinant MHM2 and MHM2Δ23-88 to RML- and 22L-associated PrP^Sc^ molecules

**Figure 6:**
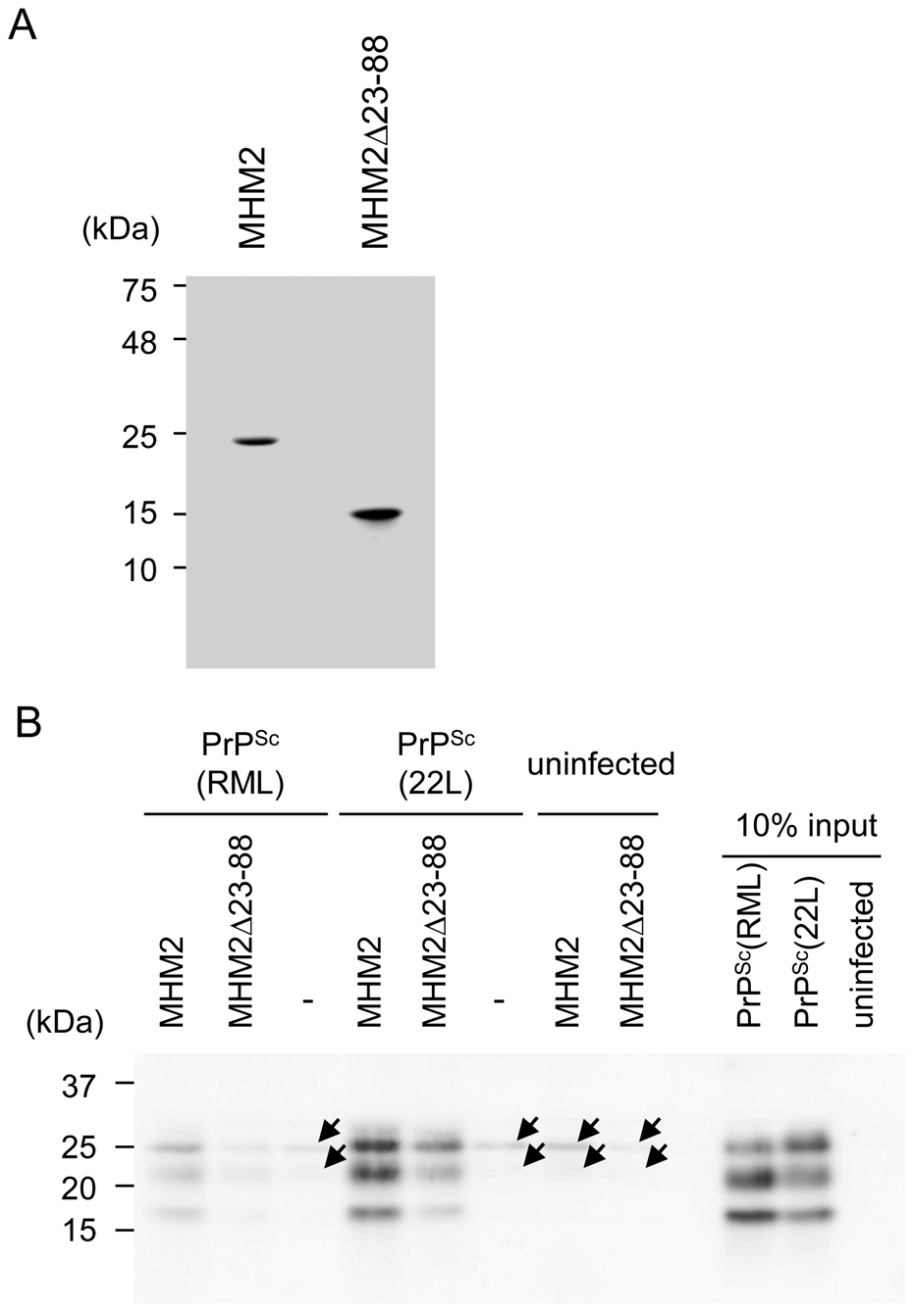
Pull-down assay for PrP^Sc^ molecules with recombinant full-length MHM2 and MHM2Δ23-88. (A) Coomassie brilliant blue staining of purified recombinant full-length MHM2 and MHM2Δ23-88. (B) PrP^Sc^(RML) and PrP^Sc^(22L) are pulled down with recombinant full-length MHM2 and MHM2Δ23-88. However, the signal for PrP^Sc^(RML) pulled down with MHM2Δ23-88 is very faint, compared to that for PrP^Sc^(22L). Arrows indicate non-specific signals that might be derived from the anti-His4 ab-Protein G beads used in this assay.

We finally investigated recombinant full-length MHM2 and MHM2Δ23-88 for their binding to PrP^Sc^ from RML-infected brain homogenates [hereafter referred to as PrP^Sc^(RML)] or PrP^Sc^ from 22L-infected brain homogenates [hereafter referred to as PrP^Sc^(22L)]. We purified recombinant full-length MHM2 and MHM2Δ23-88, both of which were tagged with 6×His (Fig. 6A). They were incubated with RML- or 22L-infected brain homogenates, in which similar amounts of PrP^Sc^(RML) and PrP^Sc^(22L) were included (Fig. 6B), and then subjected to a pull-down assay using Protein G-coupled antibodies against the 6×His tag. Full-length MHM2 pulled down both PrP^Sc^(RML) and PrP^Sc^(22L) (Fig. 6B). However, PrP^Sc^(22L) was pulled down more abundantly than PrP^Sc^(RML) (Fig. 6B). Recombinant MHM2Δ23-88 also pulled down PrP^Sc^(RML) and PrP^Sc^(22L) (Fig. 6B). However, only a tiny amount of PrP^Sc^(RML) was pulled down with MHM2Δ23-88 (Fig. 6B). In contrast, recombinant MHM2Δ23-88 pulled down a much higher amount of PrP^Sc^(22L) (Fig. 6B).

## Discussion

In the present study, we showed that intracerebral inoculation with the brain homogenate from RML-infected, terminally ill wild-type mice never caused the disease in Tg(MHM2Δ23-88)/*Prnp^0/0^* mice for up to 730 days. No MHM2^Sc^Δ23-88 was detectable in their brains. We also failed to detect any prion infectivity in the brain of a Tg(MHM2Δ23-88)/*Prnp^0/0^* mouse sacrificed at 591 days after inoculation. These results are consistent with those previously reported by others [9], [10]. In contrast, we found that Tg(MHM2Δ23-88)/*Prnp^0/0^* mice developed prion disease after intracerebral inoculation with the brain homogenate from 22L-infected, terminally ill wild-type mice, with abundant accumulation of MHM2^Sc^Δ23-88 and high prion infectivity present in their brains. These results show that MHM2Δ23-88 is converted into MHM2^Sc^Δ23-88 in Tg(MHM2Δ23-88)/*Prnp^0/0^* mice after inoculation with 22L prions. We also showed that the similarly prepared brain homogenates from terminally ill, RML- and 22L-inoculated wild-type mice contained similar infectious doses of RML and 22L prions. This excludes the possibility that unsuccessful detection of MHM2^Sc^Δ23-88 in the brains of RML-inoculated Tg(MHM2Δ23-88)/*Prnp^0/0^* mice might be due to lower infectious doses of RML prions being inoculated. It rather suggests that MHM2Δ23-88 is converted into MHM2^Sc^Δ23-88 less efficiently by RML prions than by 22L prions.

Tg(MHM2Δ23-88)/*Prnp^0/0^* mice inoculated with 22L prions developed the disease with accumulation of MHM2^Sc^Δ23-88 in their brains, indicating that MHM2^Sc^Δ23-88 lacking the N-terminal residues 23-88 is neurotoxic. However, brain spongiosis was milder in terminally ill Tg(MHM2Δ23-88)/*Prnp^0/0^* mice than in terminally ill *Prnp^0/+^* and Tg(MHM2Δ23-88)/*Prnp^0/+^* mice, suggesting that MHM2^Sc^Δ23-88 might not be fully toxic. Sonati *et al.* recently reported that the N-terminal region of PrP^C^ mediates a neurotoxic signal originating from the C-terminal globular domain bound with anti-PrP antibodies [15]. It is thus possible that the N-terminal residues might also play a role in the neurotoxicity of wild-type PrP^Sc^.

Wild-type indicator mice intracerebrally inoculated with the brain homogenate from a 22L-inoculated, terminally ill Tg(MHM2Δ23-88)/*Prnp^0/0^* mouse developed the disease significantly later than those inoculated with the brain homogenates from 22L-inoculated, terminally ill Tg(MHM2Δ23-88)/*Prnp^0/+^* and *Prnp^0/+^* mice. These results indicate that, while MHM2^Sc^Δ23-88 is infectious, it is less infectious than wild-type PrP^Sc^. Indistinguishable symptoms and PrP^Sc^ with the same PK digestion and glycosylation patterns were observed in all diseased indicator mice. These suggest that biological properties of 22L prions might not be altered even after the passage in Tg(MHM2Δ23-88)/*Prnp^0/0^* mice. Rather, the slightly longer incubation times in indicator mice inoculated with the Tg(MHM2Δ23-88)/*Prnp^0/0^* homogenate might be due to the chimeric residues in MHM2^Sc^Δ23-88. Indeed, full-length MHM2^Sc^ was shown to be slightly less infectious than wild-type PrP^Sc^ in mice overexpressing wild-type mouse PrP^C^ [9]. Alternatively, lack of the N-terminal residues in MHM2^Sc^Δ23-88 might be responsible for the longer incubation times.

*Prnp^0/0^* mice used in this study ectopically express Dpl in their brains [16], [17]. It was reported that transgenic overexpression of Dpl did not modify incubation times and brain pathologies in *Prnp^+/+^* mice infected with RML prions [18]. *Prnp^0/+^* mice with and without the ectopic expression of Dpl were also reported to display indistinguishable pathologies after infection with 301V BSE prions [19]. It is thus unlikely that Dpl affects the susceptibility of Tg(MHM2Δ23-88)/*Prnp^0/0^* mice to RML and 22L prions.

Tg(MHM2Δ23-88)/*Prnp^0/0^* mice inoculated with RML and 22L prions showed longer incubation times. Particularly, RML-inoculated Tg(MHM2Δ23-88)/*Prnp^0/0^* mice were free of the disease-specific symptoms for up to 730 days. It is known that sequence differences between PrP^C^ in recipient animals and PrP^Sc^ in an inoculum create a prion transmission barrier, causing elongation of incubation times [20]. If a prion transmission barrier is responsible for longer incubation times in primarily inoculated mice, secondarily inoculatied mice with the same genotype may result in shorter incubation times. Tg(PrPΔ23-31)/*Prnp^0/0^*, Tg(PrPΔ32-93)/*Prnp^0/0^*, and Tg(MHM2)/*Prnp^0/0^* mice primarily inoculated with RML prions were reported to show prolonged incubation times, but secondarily inoculated mice did not show shorter incubation times [9], [21], [22]. These results indicate that the N-terminal deletion or the chimeric residues in PrP^C^ does not create a prion transmission barrier for wild-type PrP^Sc^, suggesting that no prion transmission barrier is present between MHM2Δ23-88 and wild-type PrP^Sc^. Rather, we found that, in spite of overexpression of MHM2Δ23-88, MHM2^Sc^Δ23-88 was detectable in the brains of 22L-inoculated Tg(MHM2Δ23-88)/*Prnp^0/0^* mice later than wild-type PrP^Sc^ in the 22L-inoculated *Prnp^0/+^* mice, indicating that MHM2Δ23-88 converts into MHM2^Sc^Δ23-88 less efficiently than wild-type PrP^C^ into PrP^Sc^. It is therefore conceivable that the longer incubation times of Tg(MHM2Δ23-88)/*Prnp^0/0^* mice could be due to the decreased conversion of MHM2Δ23-88 into MHM2^Sc^Δ23-88. We showed that recombinant MHM2Δ23-88 pulled down PrP^Sc^(22L) and PrP^Sc^(RML) less than full-length recombinant MHM2 in a pull-down assay. Thus, the decreased conversion of MHM2Δ23-88 into MHM2^Sc^Δ23-88 might be attributable to the lower binding of PrP^Sc^(22L) and PrP^Sc^(RML) to MHM2Δ23-88.

Different strain-specific susceptibility was also reported in *Prnp^0/0^* mice transgenically expressing mouse PrP with a serine residue at codon 170, PrP-170S [23], or ovine PrP with a valine residue at codon 136, OvPrP-V136 [24]. Tg(PrP-170S)/*Prnp^0/0^* mice were highly resistant to RML and 79A prions, but susceptible to 22L and ME7 prions [23]. Only a very small number of the mice inoculated with RML and 79A prions showed brain accumulation of PrP^Sc^-170S [23]. In contrast, all mice inoculated with 22L and ME7 prions accumulated PrP^Sc^-170S in their brains [23]. Tg(OvPrP-V136)/*Prnp^0/0^* mice were susceptible to SSBP1 prions, but resistant to CH1641 prions [24]. OvPrP^Sc^-V136 was accumulated in the brains of the mice inoculated with SSBP1 prions [24]. These results suggest that strain-specific differential susceptibility in these mice is also due to different conversion efficiency of the host PrP^C^s into their PK-resistant isoforms.

The primary sequence of PrP^Sc^ is the same from different prion strains. Therefore, the different susceptibility of Tg(MHM2Δ23-88)/*Prnp^0/0^*, Tg(PrP-170S)/*Prnp^0/0^*, and Tg(OvPrP-V136)/*Prnp^0/0^* mice to different prions cannot be explained by sequence differences between PrP^Sc^ and the host PrP^C^, or that there is a prion transmission barrier. Several lines of evidence indicate that the conversion of PrP^C^ into PrP^Sc^ involves interaction of PrP^C^ with the inoculated PrP^Sc^ [25], [26]. We showed that in a pull-down assay with recombinant MHM2Δ23-88, only a tiny amount of PrP^Sc^(RML) was pulled down while a considerably higher amount of PrP^Sc^(22L) was pulled down, suggesting that different binding of MHM2Δ23-88 to PrP^Sc^(RML) and PrP^Sc^(22L) might be involved in different conversion efficiency of MHM2Δ23-88 into MHM2^Sc^Δ23-88 in RML- and 22L-inoculated Tg(MHM2Δ23-88)/*Prnp^0/0^* mice. It was reported that PrPΔ23-28 reduced the binding to PrP^Sc^(RML), and PrPΔ23-31 was insufficiently converted into PrP^Sc^Δ23-31 in mice inoculated with RML prions [21]. Miller *et al.* also reported that PrPΔ23-28 bound less PrP^Sc^(RML) and was converted very inefficiently to PrP^Sc^Δ23-28 in an *in vitro* assay [27]. MHM2Δ23-88 lacks residues 23-31, suggesting that the reduced binding of MHM2Δ23-88 to PrP^Sc^s might be partly due to the lack of residues 23-31. However, Tg(PrPΔ32-93)/*Prnp^0/0^* and Tg(MHM2)/*Prnp^0/0^* mice were also reported to have reduced susceptibility to RML prions [9], suggesting that the other deleted region(s) and the chimeric region also might be relevant to the binding of MHM2Δ23-88 to PrP^Sc^. It is thus of interest to compare the binding potential of PrP-170S and OvPrP-V136 to PrP^Sc^ from different prion strains.

The conformational selection model of prion strains also has been proposed as a mechanism to explain strain-specific susceptibility [28], [29]. This model postulates that the inoculated PrP^Sc^ selects the host PrP^C^ as a substrate for conversion due to its conformational compatibility with the host PrP^C^ [28], [29]. Conformational incompatibility between the inoculated PrP^Sc^ and the host PrP^C^ thus leads to unsuccessful or insufficient conversion of the host PrP^C^ into its PK-resistant isoform. Indeed, accumulating lines of evidence suggest that PrP^Sc^ is folded in a strain-specific conformation [30]. Nuclear magnetic resonance studies of recombinant PrPs suggest that the N-terminal domain, including the deleted residues and chimeric residues in MHM2Δ23-88, confers structural stability within the C-terminal globular domain [31], [32]. It is thus possible that MHM2Δ23-88 adopts a different conformation from that of wild-type PrP^C^, and that the adopted conformation of MHM2Δ23-88 still remains compatible with PrP^Sc^(22L), but not with PrP^Sc^(RML). The conformational selection model might also explain the different responsiveness of OvPrP-V136 and PrP-170S to different prion strains [23], [24].

It was shown that the region corresponding to the chimeric region is exposed in PrP^C^ molecules, but hidden in PrP^Sc^ molecules [33], [34], [35], and that the octapeptide repeat region of residues 51–90 is trypsin-sensitive in PrP^C^, but trypsin-resistant in PrP^Sc^ [36]. These results indicate that upon the conversion of PrP^C^ into PrP^Sc^, a marked conformational change occurs within the N-terminal domain. Interestingly, the N-terminal domain of PrP^C^ is highly flexible and displays a marked conformational heterogeneity [32], [33], [37]. It is thus also possible that lack of residues 23–88 and insertion of the chimeric residues might reduce the N-terminal conformational heterogeneity of MHM2Δ23-88, and that the reduced conformational heterogeneity might render MHM2Δ23-88 resistant to RML but still susceptible to 22L prions.

The N-terminal domain was shown to be important for internalization of PrP^C^ [38]. The conversion of PrP^C^ into PrP^Sc^ has been suggested to occur on the cell surface and/or along the endocytic pathway to lysosomes [39], [40]. Thus, defective internalization of MHM2Δ23-88 might lead to the insufficient conversion of MHM2Δ23-88 to MHM2^Sc^Δ23-88. However, it remains unknown how internalized PrP^C^ can undergo strain specific conversion.

Tg(MHM2Δ23-88)/*Prnp^0/+^* mice showed shorter incubation times than *Prnp^0/+^* mice after inoculation with RML prions. This is consistent with the previously reported results [9], [10]. However, incubation times were not shortened in Tg(MHM2Δ23-88)/*Prnp^0/+^* mice inoculated with 22L prions. It was shown that the co-expressing wild-type PrP^C^ *trans*-acts MHM2Δ23-88 to assist its conversion into MHM2^Sc^Δ23-88 after infection with RML prions, by demonstrating that RML-inoculated Tg(MHM2Δ23-88)/*Prnp^0/+^* mice produced MHM2^Sc^Δ23-88 in their brains [10]. We also observed that the conversion of MHM2Δ23-88 into MHM2^Sc^Δ23-88 was enhanced in RML- and 22L-inoculated Tg(MHM2Δ23-88)/*Prnp^0/+^* mice. Interestingly, we found that, in contrast to the conversion of MHM2Δ23-88 into MHM2^Sc^Δ23-88 being enhanced, the conversion of wild-type PrP^C^ into PrP^Sc^ was decelerated in Tg(MHM2Δ23-88)/*Prnp^0/+^* mice inoculated with RML and 22L prions. These results indicate that the *trans*-action by PrP^C^ and the *trans*-inhibition by MHM2Δ23-88 reciprocally affect the production of wild-type PrP^Sc^ and MHM2^Sc^Δ23-88 in RML- and 22L-inoculated Tg(MHM2Δ23-88)/*Prnp^0/+^* mice. Thus, investigation of detailed accumulation kinetics of wild-type PrP^Sc^ and MHM2^Sc^Δ23-88 in the brains of RML- and 22L-inoculated Tg(MHM2Δ23-88)/*Prnp^0/+^* mice would be helpful to understand the strain-specific disease progression in Tg(MHM2Δ23-88)/*Prnp^0/+^* mice.

What is the mechanism for MHM2Δ23-88 to decelerate the conversion of wild-type PrP^C^ into PrP^Sc^? Two possibilities have been proposed for the *trans*-action of PrP^C^ on the conversion of MHM2Δ23-88 to MHM2^Sc^Δ23-88 [10]. One is that PrP^C^ might bind to MHM2Δ23-88 and then promote the conversion of MHM2Δ23-88 into MHM2^Sc^Δ23-88. In this scenario, MHM2Δ23-88 having increased conversion competence with help from the co-expressing PrP^C^ might compete with the co-expressing PrP^C^ for an as yet unidentified factor(s) important for the conversion, such as a conjectural molecule protein X, thereby decelerating the conversion of PrP^C^ into PrP^Sc^. The other is that PrP^C^ is first converted into PrP^Sc^ and the nascent PrP^Sc^ then acts on MHM2Δ23-88 to convert it into MHM2^Sc^Δ23-88. In this case, since MHM2Δ23-88 converts into MHM2^Sc^Δ23-88 very slowly, PrP^Sc^ participating in the conversion of MHM2Δ23-88 into MHM2^Sc^Δ23-88 could not engage in other conversion events until MHM2Δ23-88 is converted into MHM2^Sc^Δ23-88, ultimately causing the decreased conversion of PrP^C^ into PrP^Sc^. Elucidation of the *trans*-action of PrP^C^ on the conversion of MHM2Δ23-88 into MHM2^Sc^Δ23-88 and the *trans*-inhibition of MHM2Δ23-88 on the conversion of wild-type PrP^C^ into PrP^Sc^ would be worthy for understanding the conversion mechanism of PrP^C^ into PrP^Sc^.

## Supporting Information

Figure S1 **Higher magnification images of HE-stained brain sections from different genotypic mice inoculated with prions.** (A) Spongiosis is milder in the cerebral cortex, hippocampus, thalamus and cerebellum from 22L-inoculated, terminally ill Tg(MHM2Δ23-88)/*Prnp^0/0^* mice than in 22L-inoculated, terminally ill *Prnp^0/+^* and Tg(MHM2Δ23-88)/*Prnp^0/+^* mice. (B) Spongiosis is observed in the cerebral cortex, hippocampus, thalamus and cerebellum from RML-inoculated, terminally ill *Prnp^0/+^* and Tg(MHM2Δ23-88)/*Prnp^0/+^* mice, but not from RML-inoculated, symptom-free Tg(MHM2Δ23-88)/*Prnp^0/0^* mice. Scale bar, 100 µm.
(TIF)
