# Supplementary figures and images for: Mouse-Hamster Chimeric Prion Protein (PrP) Devoid of N-Terminal Residues 23-88 Restores Susceptibility to 22L Prions, but Not to RML Prions in PrP-Knockout Mice
Source: PLoS One. 2014 Oct 16;9(10):e109737. doi: 10.1371/journal.pone.0109737 (PMC4199594; doi:10.1371/journal.pone.0109737)

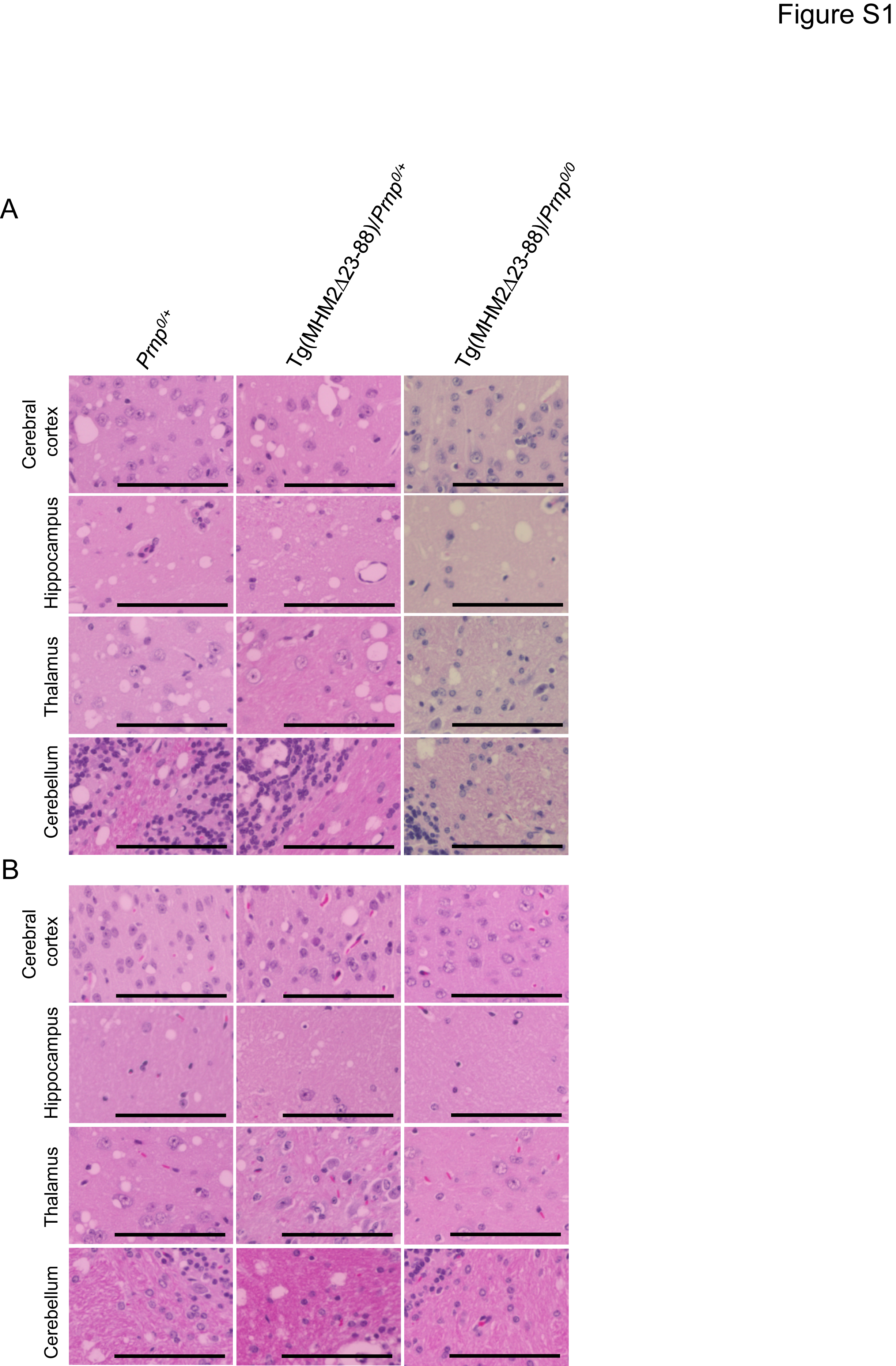

Supplement: Figure S1 — Higher magnification images of HE-stained brain sections from different genotypic mice inoculated with prions. (A) Spongiosis is milder in the cerebral cortex, hippocampus, thalamus and cerebellum from 22L-inoculated, terminally ill Tg(MHM2Δ23-88)/Prnp0/0 mice than in 22L-inoculated, terminally ill Prnp0/+ and Tg(MHM2Δ23-88)/Prnp0/+ mice. (B) Spongiosis is observed in the cerebral cortex, hippocampus, thalamus and cerebellum from RML-inoculated, terminally ill Prnp0/+ and Tg(MHM2Δ23-88)/Prnp0/+ mice, but not from RML-inoculated, symptom-free Tg(MHM2Δ23-88)/Prnp0/0 mice. Scale bar, 100 µm. (TIF) [file pone.0109737.s001.tif]
